# Predictors and grade level trends of school day physical activity achievement in low-income children from the U.S.

**DOI:** 10.1016/j.pmedr.2015.10.002

**Published:** 2015-10-21

**Authors:** Ryan D. Burns, Timothy A. Brusseau, Yi Fang, Rachel S. Myrer, You Fu, James C. Hannon

**Affiliations:** a University of Utah, Department of Exercise and Sport Science, 250 S. 1850 E., HPER North, RM 241, Salt Lake City, UT 84112, USA; b Salt Lake City School District, 440 East 100 South Salt Lake City, UT 84111, USA; c University of Nebraska Kearney, Kinesiology and Sports Sciences Department, 905 West 25th Street, Kearney, NE 68849, USA; d West Virginia University, College of Physical Activity and Sport Sciences, P.O. Box 6116, 375 Birch St., Morgantown, WV 26505-6116, USA

**Keywords:** Accelerometers, Health-related fitness, Pedometers, Sedentary times

## Abstract

The achievement of recommended levels (≥ 30 min/day) of school moderate-to-vigorous physical activity (MVPA) is paramount to decrease risk of chronic disease in children from low-income families. The purpose of this study was to examine the predictors and grade-level trends of school day MVPA achievement in low-income children. Data were collected during the Fall of 2014 on 1232 children (Mean age = 8.8 ± 1.6 years; 625 girls, 607 boys) recruited from three low-income schools from the state of Utah in the U.S. Children wore pedometers for one school week and a stratified random subsample (*n* = 533) also wore accelerometers to record sedentary time and MVPA. Generalized linear mixed models were employed to calculate odds ratios for achieving school MVPA standards (≥ 30 min/day) from various predictors and to determine odds of achievement across grade levels, accounting for school and classroom clustering. Odds of meeting MVPA standards were 3 times greater if a student achieved at least 6000 steps during the school day (*p* < 0.01), and were 55% lower for every 1% increase in sedentary time (*p* < 0.001). Older children had 26% lower odds of meeting the recommended levels of MVPA compared to children in an immediately younger grade level (*p* < 0.05). A significant proportion of MVPA variance was explained by classroom and school affiliation (Rho = 0.09 to 0.54, *p* < 0.001). Daily steps, sedentary times, grade level, and classroom and school affiliation associate with school MVPA achievement in low-income children.

## Highlights

• Pedometer steps and sedentary time are predictors of school day MVPA achievement.
• The prevalence of children achieving MVPA standards is lower in older grades.
• Classroom and school affiliation explained significant variance in school day MVPA.

## Introduction

The health benefits of optimal levels of moderate-to-vigorous physical activity (MVPA) are numerous in school-aged children (Boreham and Riddoch, 2001, Mitchell et al., 2013, Trudeau and Shephard, 2008, Yli-Piipari et al., 2012). Optimal MVPA has been linked to decreases in early onset risk factors for cardio-metabolic disease and has a protective effect on excess weight gain during the developmental years (Boddy et al., 2014, Duncan et al., 2010). Because a significant portion of the week is spent in school for most children in the U.S., research focusing on improving school physical activity behaviors has become imperative. Although the majority of the school day is spent in sedentary behaviors, there are specific times where children have opportunities to engage in active play to increase their daily physical activity levels. These include leisure times such as before school, during recess, classroom activity breaks, physical education (PE), and after school (Ridgers et al., 2006, McKenzie et al., 2010). Despite the opportunities for school-aged children to engage in active play, many children do not fully participate in leisure time physical activity and thus fail to meet the recommended 30 min of MVPA during school hours and 60 min during the entire day (Troiano et al., 2008, U.S. Department of Health and Human Services, 2008, Dumith et al., 2011).

All populations of children can benefit from achieving recommended levels of MVPA during school hours, however children from low-income families may especially be of benefit (Gordon-Larsen et al., 2006). Low-income children may not have sufficient access to participate in active play before or after school hours, have parents with poor physical activity behaviors, have low self-efficacy for physical activity, and may have a stronger disposition to early onset chronic disease risk factors if of Hispanic ethnicity because of genetic and environmental factors (Lampard et al., 2013, Butte et al., 2007, Messiah et al., 2013). Indeed, it has been shown that low socio-economic status (SES) children display lower levels of MVPA compared to higher SES children (Borraccino et al., 2009, Ferreira et al., 2007).

Despite numerous research studies examining correlates of MVPA in children (Carnethon et al., 2005, Eisenmann, 2004, Eisenmann et al., 2007, Dencker and Andersen, 2011), there has been limited research focusing on samples of low-income elementary school-aged children in the U.S. Because of the evidence showing differences in MVPA levels between low and high SES children during school, factors that relate to school MVPA may also be different (Young et al., 2007, Turner et al., 2010). Therefore, identifying the predictors of meeting recommended school MVPA levels (≥ 30 min MVPA/day) can help researchers and practitioners devise intervention strategies that can improve odds of student achievement. Additionally, identifying the grade level trends in meeting the recommended levels of MVPA in low-income children is also important, as MVPA tends to decrease across grade levels in the general pediatric population and this trend may be exacerbated in lower SES populations (Pate et al., 2002, Centers for Disease Control and Prevention, 2003). Indeed, grade level trends using age- and sex-specific health-related fitness criterion-referenced standards and school day MVPA cut-points has not been reported in the current literature. Thus quantifying decreases in health-related fitness and school day MVPA achievement across elementary grade levels in low-income children will provide important information.

Potential correlates of MVPA achievement include a child's aerobic capacity and BMI (health-related fitness) in addition to other physical activity correlates such as sedentary time and pedometer steps. These parameters are measurable, quantifiable, and modifiable variables that can be targeted by researchers and practitioners engaging in intervention work (Centers for Disease Control and Prevention, 2013).Although these measures may relate to all children's school day MVPA, the cross-sectional relationships in low-income children have yet to be explored and quantified. Therefore, the purpose of this study was to examine the predictive relationships between meeting recommended school day MVPA with sedentary times, pedometer step counts, and health-related fitness in low-income children from the 1st through 6th grades. A secondary aim was to model the cross-sectional grade level trends in health-related fitness and MVPA achievement. The authors wanted to examine what the specific predictive relationships were between a child meeting recommended MVPA levels using a criterion measure (i.e. accelerometers) with pedometer step counts, sedentary times, and two domains of health-related fitness including body composition and aerobic capacity. The authors also wanted to explore how meeting optimal and recommended levels of each of these parameters differ across grade levels in low-income children.

## Methods

### Participants

Participants were a convenience sample of 1232 school-aged children recruited from three low-income elementary schools from the state of Utah in the U.S. The students were recruited based on being enrolled in a school district that received funding from the U.S. Department of Education in the form of a Carol White Physical Education Program (PEP) Grant. Data within the current study were from the measures collected at baseline, before implementation of any programming. All students within the three schools participated in the study. The majority of the sample was of Hispanic/Latino ethnicity (60.60%), followed by Pacific Islander (13.70%), Caucasian (10%), African American (7.80%), Asian (3.50%), and approximately 4% was characterized as “Other”. Given the data from the State Department of Education and the school district website, 91%, 95%, and 96% of the children at each of the three schools were from low-income families during the 2014−2015 school year (http://www.schools.utah.gov/data/). Children were recruited from the 1st−6th grades. The mean age of the sample was 8.8 ± 1.6 years and the average class size was 22.4 ± 2.5 children. Written assent was obtained from the students and consent was obtained from the parents prior to data collection. The University of Utah Institutional Review Board approved the protocols employed in this study.

### Measures

Physical activity was measured using Yamax DigiWalker CW600 pedometers (Tokyo, Japan) and ActiGraph wGT3X-BT triaxial accelerometers (Pensacola, FL, USA). Each student in the sample (*N* = 1232) wore a pedometer for one school week, and a stratified randomly selected sub-sample of 533 students (277 girls, 256 boys) also wore accelerometers. Only a randomly selected sub-sample of children wore accelerometers because of limited equipment availability. One to two classrooms per grade per school were randomly selected to wear accelerometers in addition to pedometers. The devices were worn for one school week (5 consecutive school days; Monday through Friday) between the hours of 8am and 3pm with no included non-wear time (i.e. time when students take off the device). Both devices were worn on the hip at the level of the iliac crest above the right knee with pedometers worn on the left hip and accelerometers worn on the right hip. Each pedometer and accelerometer was given an identification number and assigned to a student with the corresponding identifier.

Yamax DigiWalker models have been shown to provide an accurate recording of steps within ± 3% of actual steps (Schneider et al., 2003), and have been shown to be a valid measure of free-living physical activity (Crouter et al., 2003). Accelerometer data were recorded in 5-second epochs at 100 Hertz and then processed using Evenson et al. (2008) cut-points, as recommended by Trost et al. (2011) based on strong criterion-referenced energy expenditure agreement using indirect calorimetry. A valid day was recorded as accelerometer data for at least six hours of a seven-hour school day. Participants were included in the analysis if they had recorded data for at least 4 valid days of the school week to ensure that the devices were worn for the majority of the school week (96% of the sub-sample; 512 children). Analyzed accelerometer data included the percent of total wear time in sedentary time (%sedentary) and average daily time in MVPA (in minutes; daily MVPA). MVPA was recorded in minutes in order to stratify the variable into a binary classifier based on current physical activity recommendations (see Data Processing). The ActiLife 6.11.5 software program (Pensacola, FL, USA) was used to initialize, download, process, and store accelerometer data.

Aerobic capacity was estimated using the 15-meter (Grades 1−3) and 20-meter (Grades 4−6) progressive aerobic cardiovascular endurance run (PACER), administered during each student's PE class. The PACER was conducted on a marked gymnasium floor with background music provided by a compact disk. Each student was instructed to run from one floor marker to another floor marker across a 20-meter distance within an allotted time frame. The allotted time given to reach the specified distance incrementally shortened as the test progressed. If the student twice failed to reach the other floor marker within the allotted time frame, the test was terminated (Meredith and Welk, 2010). Even though data were collected across all grade levels, proper technique and enjoyment of the test were emphasized over performance in younger children from the 1st to 3rd grades, given the recommendations in the FITNESSGRAM manual (Meredith and Welk, 2010). The final score was recorded in laps. Estimated VO_2 Peak_ was then calculated from PACER laps using a validated prediction algorithm currently used by FITNESSGRAM using laps and age predictor variables. Weight was measured using a portable medical scale (BD-590; Tokyo, Japan) and height was measured using a portable stadiometer (Seca 213; Hanover, MD, USA). BMI was calculated using standard procedures by taking a students' weight in kilograms divided by the square of his or her height in meters.

### Procedures

Data were collected and analyzed during Fall semester 2014. Health-related fitness and physical activity measures were collected during separate weeks at each of the three schools. During PE, students were randomly assigned to three stations and completed assessments at each respective station. The three stations included the PACER, anthropometric assessments (i.e. height and weight), and a station to complete demographic questionnaires that asked students to specify their age, grade, sex, and ethnicity. Students completed the questionnaire instead of parents to maintain a high response rate. A trained member of the research team (PI, research associate, or graduate research assistant) aided children on answering the demographic questions and collected all other measures to maintain testing accuracy and consistency. Pedometers and accelerometers were administered no less than one week and no more than three weeks following health-related fitness testing at each school and data were collected using the procedures described previously.

### Data processing

Physical activity data from pedometers and accelerometers were stratified into a binary classification scheme having levels of “meeting” and “not meeting” school day standards for MVPA. The pedometer step count cut-point was set at 6000 steps per school day (one half of the 12,000 steps per day recommended by Colley et al., 2012). A dichotomous variable was calculated from accelerometer data using an average MVPA per school day cut-point of ≥ 30 min per day recommended by the Institute of Medicine (2013). Aerobic capacity and body composition data were also stratified into a binary classification scheme using FITNESSGRAM's age and sex specific criterion-referenced standards for estimated VO_2 Peak_ and BMI, respectively. The standards for BMI and VO _Peak_ are age and sex specific, validated against metabolic syndrome criteria, and is currently the standard used for fitness assessment in the U.S. (Welk et al., 2011a) The two levels for the aerobic capacity and BMI variables represented the healthy fitness zone (HFZ or “met standard”) and a needs improvement (NI or “not met standard”) classifier by combining the two NI sub-zones used in the FITNESSGRAM program (http://www.fitnessgram.net).

### Statistical analysis

The main analysis consisted of employing generalized linear mixed models (GLMM; logit link function) using classroom level and school level random intercepts to calculate odds ratios for meeting school day MVPA standards. GLMMs were used to account for the clustering of individual measurements within classrooms (level 2), as there were a total of 70 classrooms among the three schools, and the clustering of classrooms within schools (level 3). Predictors included the achievement of the age and sex specific VO_2 Peak_ and BMI standards (dichotomous variables), step count achievement (dichotomous variable), and percent of total wear time in sedentary behavior. Predictors were entered into their own separate model because of possible multicollinearity among the parameters. Because of the use of multiple models, the alpha level was adjusted using the Bonferroni method to determine statistical significance. Sex and age were entered into each model to control for a possible confounding effect. Results were reported as adjusted odds ratios for each of the aforementioned predictors.

To model the cross-sectional grade-level trends in the aforementioned dichotomous parameters, separate GLMM models (logit link function) with school level random intercepts were employed. These analyses were used to account for the clustering of grade levels within each of the three schools. The classroom cluster was omitted because grade levels are not clustered within classrooms. Separate models used dependent variables of HFZ VO_2 Peak_ achievement, HFZ BMI achievement, daily step count achievement, and daily MVPA achievement. The predictor variable in each of the models was grade level. Results were reported as grade level adjusted odds ratios, which was the average change in odds in cohorts of children separated by one grade level. The sex of the child was entered into each model to control for possible confounding. Chi-square tests were used to validate the use of GLMMs by examining the likelihood ratio null hypothesis that the fraction of variance due the differences among the panels (clusters) is equal to zero (i.e. Rho = 0; using STATA's xtlogit). An a priori power analysis for logistic regression revealed that approximately 459 students were needed to achieve 80% power given a medium effect size. Therefore, it was determined that each of the aforementioned analyses had an adequate sample size. Less than 5% of the sample was missing for each variable, therefore it was unlikely that the parameters have been biased in a substantial manner. Alpha level was set at *p* ≤ 0.01 to adjust for multiple outcomes and all analyses were carried out using STATA 13.0 statistical software package (College Station, TX, USA).

## Results

The descriptive data for health-related fitness and physical activity are presented in Table 1 for the total sample and within each sex and grade level. Table 2 shows the adjusted odds ratios for meeting school day MVPA standards across the dichotomous and continuous predictors. The odds of meeting daily MVPA standards were 3.0 times greater if a student achieved an average of at least 6000 steps during the school day (*p* < 0.01). Also, the odds of a student meeting daily MVPA standards were 55.1% lower for every 1% increase of total time spent in sedentary time (*p* < 0.001). Being classified in the HFZ for either VO_2 Peak_ or BMI did not associate with any change in odds. The fraction of variance explained by the differences among classroom and school affiliation ranged from Rho = 25% to 46% (*p* < 0.001).

**Fig. 1:**
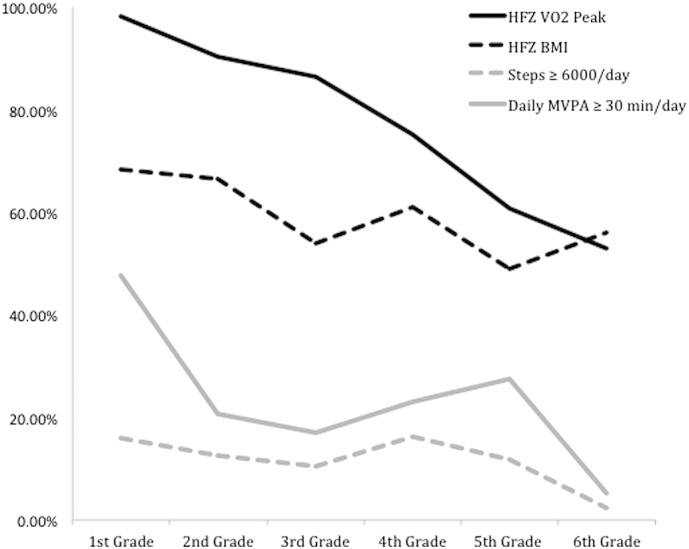
Mean health-related fitness and physical activity percent standard achievement across grade levels. *Note:* All data were collected during Fall semester 2014 from three schools located from the state of Utah in the U.S.

Table 3 shows the grade level parameter estimates (cross-sectional trends) from the GLMMs using various criterion (dependent) variables. For cohorts separated by one grade level, there was a 51.7%, 17.5%, and 26.0% lower odds of achieving the HFZ for VO_2 Peak_, the HFZ for BMI, and meeting the daily standard for MVPA, respectively (*p* < 0.01). Fig. 1 displays a line graph showing the grade-level trends in meeting standards for health-related fitness and physical activity in separate cohorts of students from the 1st through 6th grades. The fraction of variance explained by differences among school affiliation ranged from Rho = 8% to 19% (*p* < 0.001).

## Discussion

The purpose of this study was to examine the predictors and grade-level trends of school day MVPA achievement in elementary school-aged children from low-income families. The results indicate that steps per school day, sedentary time, and a child's grade level associate with odds of achieving school MVPA standards. Additionally, a significant proportion of the variance in odds of school day MVPA achievement was explained by classroom and school affiliation. Although the results are consistent with findings using other similar aged populations of youth (Kahn et al., 2008, Nader et al., 2008, Brodersen et al., 2007), what is potentially unique was the use of a large sample of school-aged children enrolled exclusively in low-income (Title I) schools. Despite the novelty, comparisons were not made with higher SES children therefore it is unknown whether the results obtained would differ between low and high-SES cohorts. Low-income children have additional burdens for achieving recommended MVPA and its associated health benefits (Gordon-Larsen et al., 2006, Lampard et al., 2013, Butte et al., 2007, Messiah et al., 2013). Steps per school day, sedentary times, grade level, and classroom and school affiliation associated with the odds of achieving MVPA standards and should be a priority in future intervention strategies targeting low-income children.

Accelerometers are often used as the criterion measure for physical activity surveillance and assessment (Welk, 2002). However, their use in large samples is limited by cost and accessibility (Tudor-Locke and Bassett, 2004). The results from this study support that pedometer step counts associate strongly to the odds of a student achieving MVPA standards. It has been recently suggested that children and adolescents should accumulate at least 12,000 steps per day for health benefits (Trost et al., 2011). Although there is no consensus for an optimal school day step count standard (Tudor-Locke and Bassett, 2004), a cut-point of 6000 steps is defensible, as it can be assumed that approximately half of a student's waking hours are during school hours. The results show that achieving this cut-point associated with approximately 3-fold greater odds of achieving at least 30 min per day in MVPA during school. Therefore, for researchers or practitioners engaging in intervention work, increasing daily steps, especially above 6000 per school day, is a reasonable objective in lieu of accelerometer measurement, which is more cumbersome to administer to large populations of children in school settings.

Sedentary time was also significantly associated with MVPA achievement. Specifically, a 1% increase in school sedentary time decreased the odds of a student achieving school day MVPA standards by approximately 55%. Practically, over a school week of 2100 min (7 h school days, 5 days per week), a 1% increase in sedentary behavior is 21 min per week, or approximately 4 min per school day. This is a significant finding for the practical application of decreasing sedentary time in school-aged children and its possible implications on MVPA. Interventions implementing classroom activity breaks such as the “TAKE 10!” program (http://www.take10.net/programmain) can easily attenuate sedentary behaviors across the school week by 1% in order to increase odds of a child meeting MVPA standards (Goh et al., 2014). Additionally, sedentary times have been shown as an independent risk factor for chronic disease incidence (Biswas et al., 2015). Indeed, there have recently been numerous research studies linking excess sedentary behavior to health risk in both pediatric and adult populations (Gordon-Larsen et al., 2002, Katzmarzyk, 2010, Thorp et al., 2011, Owen et al., 2011). The findings from the current study suggest that a relatively small decrease in sedentary time during school hours can significantly improve odds of meeting school MVPA, which attenuates health risk and improves health outcomes (Boreham and Riddoch, 2001, Trudeau and Shephard, 2008, Yli-Piipari et al., 2012).

Past research has established that children's MVPA significantly decreases with age (Anderssen et al., 2005, Janz et al., 2000), however the results from the current study are especially concerning. For nearly all parameters, the odds of achieving optimal health-related fitness and MVPA were drastically lower in older grade cohorts compared to younger grade cohorts. The results support previous work, however the decreases in low-income youth may be more problematic when also considering that the data were consistently lower than national averages at each grade level (Welk et al., 2011b, Centers for Disease Control, 2014, Jin and Jones-Smith, 2015); however it is not clear if the results obtained from this study are primarily attributable to the low-income characteristics of this sample or to the state from which the data were collected. Despite this uncertainty, it is important for future interventions to attenuate these declines in all population of children to improve health outcomes as they track through adolescence and into adulthood.

An unexpected finding from the current study is that a significant portion of the variance in MVPA achievement was explained by classroom and school affiliation. Indeed, in some of the prediction models classroom and school affiliation accounted for more than 50% of the variance in the odds of a child achieving at least 30 min of MVPA during school. This supports that interventions hoping to improve school MVPA in all populations of children should focus changing the school and, more specifically, the classroom environment (i.e. at the teacher level) in order to improve the odds that a child can meet recommended daily guidelines. Past research has consistently shown that contextual factors relates to children's MVPA and health-related fitness (Czerwinski et al., 2015). These factors include but are not limited to school's built-in environment such as access to playground equipment and open play space, physical characteristics of school gymnasiums, and the home environment (Loprinzi et al., 2012). Physical activity behaviors and health-related fitness of classroom teachers also play a significant role in the MVPA patterns of the general pediatric population (Centers for Disease Control and Prevention, 2013). Unfortunately, these factors were not explored in the current study and have a high priority for future work examining correlates of MVPA in low-income children.

## Study limitations

There are limitations that must be considered. First, the sample consisted of three low-income schools from the state of Utah in the U.S. with data collected during Fall semester, therefore the external validity of the results is questionable if generalized to other regions or during Spring semester. Second, this study used a cross-sectional descriptive research design; therefore the longitudinal effects at the student level were not addressed and no causal relationships could be established. Third, VO_2 Peak_ and BMI achievement did not associate with MVPA achievement. This may be an issue of research design as VO_2 Peak_ and BMI may be outcomes of the longitudinal effect of MVPA achievement and thus the relationships cannot be adequately addressed using cross-sectional designs (Czerwinski et al., 2015). Finally, because a significant proportion of the variance was explained by classroom and school affiliation, factors such as each school's built-in environment and factors associated with schoolteachers such as their own MVPA, efficacy for physical activity, and motivation to implement physical activity breaks during class time may have displayed significant relationships. As stated previously, these parameters were not collected and should be a priority in future research using samples of low-income children.

## Conclusions

In conclusion, pedometer step counts, percent of total time in sedentary time, and classroom and school affiliation significantly associated with the odds of achieving daily MVPA during school hours in low-income children. Furthermore, there were significantly lower levels of health-related fitness and MVPA achievement in older cohorts compared to younger cohorts. Although VO_2 Peak_ and BMI achievement did not associate with the MVPA parameters, the lack of association may have been limited by the cross-sectional research design. It is imperative that future research examines other possible correlates of school day MVPA that may be unique to low-income children. Regardless, this study provides insights on the predictors and grade-level trends of school MVPA in low-income children and provides information on the variables that researchers and practitioners should target in order to improve MVPA achievement in a burdened population of America's youth.

## Conflict of interest statement

The authors declare that there are no conflicts of interest.

## Figures and Tables

**Table 1:** Descriptive statistics for the total sample and within sex and grade groups (means and standard deviations).

	Total sample	Girls	Boys	1st grade	2nd grade	3rd grade	4th grade	5th grade	6th grade
VO_2 Peak_ (ml kg^− 1^ min^− 1^) (*N* = 1232)	44.32 ± 5.14	43.91 ± 4.83	44.76 ± 5.42	45.06 ± 3.02	45.30 ± 4.21	45.79 ± 5.40	44.76 ± 4.96	42.81 ± 4.93	44.07 ± 8.25
BMI (kg m^2^) (*N* = 1232)	18.39 ± 5.00	18.32 ± 4.89	18.37 ± 5.11	16.71 ± 3.39	17.40 ± 3.65	18.90 ± 3.95	19.08 ± 4.42	21.78 ± 7.73	20.66 ± 4.85
Average steps (*N* = 1232)	4285.99 ± 1535.48	4149.20 ± 1444.43	4442.23 ± 1620.92	4562.91 ± 1480.80	4291.72 ± 1571.15	4173.58 ± 1500.48	4529.06 ± 1599.31	4266.67 ± 1444.71	3630.61 ± 1407.31
%Sedentary (*n* = 512)	92.08 ± 3.56	92.28 ± 3.45	91.86 ± 3.66	89.73 ± 3.51	92.11 ± 3.45	92.16 ± 2.67	93.04 ± 4.02	92.44 ± 2.83	93.89 ± 3.27
Daily MVPA minutes (*n* = 512)	22.53 ± 12.62	20.94 ± 11.54	24.28 ± 13.51	29.51 ± 13.17	21.75 ± 12.54	21.96 ± 10.28	20.23 ± 14.17	23.15 ± 11.25	15.69 ± 8.37

*Note*: BMI stands for body mass index; %Sedentary is the percent of total wear time in sedentary time. All data were collected during Fall semester 2014 from three schools located from the state of Utah in the U.S.

**Table 2:** Adjusted odds ratios (ORs) from generalized linear mixed models.

	Odds ratio	95% C.I.	*p*-Value	Rho
*Criterion is MVPA ≥ 30 min/school day*				
HFZ VO_2 Peak_	0.88	0.37, 2.31	0.750	**0.46**
HFZ BMI	1.01	0.52, 1.82	0.982	**0.54**
Steps ≥ 6000/school day	**3.02**	1.41, 7.08	**< 0.001**	**0.42**
%Sedentary	**0.45**	0.34, 0.59	**< 0.001**	**0.25**

*Note*: Rho is the proportion of variance explained by classroom affiliation and school affiliation; HFZ stands for the Healthy Fitness Zone; BMI stands for body mass index; %Sedentary is the percent of total wear time in sedentary time; boldface indicates statistical significance (*p* < 0.001). All data were collected during Fall semester 2014 from three schools located from the state of Utah in the U.S.

**Table 3:** Adjusted grade level odds ratios from generalized linear mixed models.

Criterion	Grade level odds ratio	95% C.I.	*p*-Value	Rho
% HFZ VO_2 Peak_	**0.48**⁎⁎	0.45, 0.51	**< 0.001**	**0.09**⁎⁎
% HFZ BMI	**0.82**⁎⁎	0.78, 0.87	**< 0.001**	**0.08**⁎⁎
% Steps ≥ 6000/school day	1.02	0.92, 1.14	0.665	**0.08**⁎⁎
% MVPA ≥ 30 min/school day	**0.73**⁎	0.55, 0.96	**0.020**	**0.19**⁎⁎

*Note*: Rho is the proportion of variance explained by school affiliation; HFZ stands for the Healthy Fitness Zone. BMI stands for body mass index; boldface indicates statistical significance. All data were collected during Fall semester 2014 from three schools located from the state of Utah in the U.S.

⁎ *p* < 0.05.

⁎⁎ *p* < 0.001.
